# Supplementary material for: scMAR-Seq: a novel workflow for targeted single-cell genomics of microorganisms using radioactive labeling
Source: mSystems. 2023 Nov 20;8(6):e00998-23. doi: 10.1128/msystems.00998-23 (PMC10734494; doi:10.1128/msystems.00998-23)
Supplement: Supplemental material — Supplemental tables, figures, and movie legends. [file msystems.00998-23-s0001.docx]

Supporting Information for

**scMAR-Seq: A novel workflow for targeted single-cell genomics of microorganisms using radioactive labelling**

Hao-Yu Lo^1,2^, Konstantin Wink^3^, Henrike Nitz^2^, Matthias Kästner^2^, Detlev Belder^3^, Jochen A. Müller^1,2*^, Anne-Kristin Kaster^1*^

^1^ Institute for Biological Interfaces (IBG-5), Karlsruhe Institute of Technology, Eggenstein-Leopoldshafen, Germany

^2^ Department of Environmental Biotechnology, Helmholtz Centre for Environmental Research - UFZ, Leipzig, Germany

^3^ Institute for Analytical Chemistry, Leipzig University, Leipzig, Germany

* Correspondence:

Institute for Biological Interfaces (IBG 5),

Karlsruhe Institute of Technology,

Hermann-von-Helmholtz-Platz 1

76344 Eggenstein-Leopoldshafen, Germany

Email: [kaster@kit.edu](http://kaster@kit.edu)

**This file includes:**

**Table S1**. Overview of alginate gelation kinetics utilized in microfluidic systems with their strengths and weaknesses

**Table S2**. Summary statistics of SAGs from microcapsules

**Figure S1.** (A) The photomask for photolithography on a 4-inch silicon wafer and (B) the geometry details of the design pattern. (C) The microfluidic chip was fabricated in-house.

**Figure S2.** The modified radiographic procedures for applying MAR on alginate microcapsules.

**In addition, Supplementary Movies S1 and S2 are available on the mSystems website.**

**Movie S1.** PDMS microfluidic chip in operation for scMAR-Seq

**Movie S2.** Oil droplets rolling along the microchannel towards the outlet port of the PDMS chip

**Table S1.** Overview of alginate gelation kinetics utilized in microfluidic systems with their strengths and weaknesses

|  | **Solutions 1**  **(Gelling precursor)** | | | | **Solution 2**  **(Exchange precursor)** | | | | **Microcapsule rigidity** | **Pre-mature clogging or**  **coalescence** |
| --- | --- | --- | --- | --- | --- | --- | --- | --- | --- | --- |
|  | ion (mM) | | EDTA (mM) | Alginate % (wt) | ion (mM) | | EDDA (mM) | Alginate % (wt) |  |  |
| CLEX T1 | Ca^2+^ | 84 | 84 | 0.6 | Zn^2+^ | 84 | 84 | 0.6 | Good | - |
| CLEX T2 | Ba^2+^ | 84 | 84 | 0.6 | Zn^2+^ | 84 | 84 | 0.6 | - | Pre-mature clog |
| CLEX T3 | Ba^2+^ | 94 | 94 | 0.5 | Zn^2+^ | 94 | 94 | 0.6 | - | Pre-mature clog |
| CLEX T4 | Ba^2+^ | 94 | 94 | 0.4 | Zn^2+^ | 125 | 62.5 | 0.25 | Poor | Coalescence |
| CLEX T5 | Ba^2+^ | 94 | 94 | 0.3 | Zn^2+^ | 125 | 62.5 | 0.5 | Poor | Coalescence |
| CLEX T6 | Ba^2+^ | 94 | 94 | 0.25 | Zn^2+^ | 125 | 125 | 0.5 | Good | - |
| CLEX T7 | Ba^2+^ | 94 | 94 | 0.25 | Zn^2+^ | 300 | 150 | 0.5 | Good | Pre-mature clog |
| CLEX T8 | Ba^2+^ | 94 | 94 | 0.25 | Zn^2+^ | 300 | 300 | 0.8 | Excellent | - |

**Table S2**. Summary statistics of SAGs from microcapsules

|  | **FACS**  **sorted** | **WGA-X amplified**  number (percent of sorted) | **Successful libraries**  number (percent of WGA-X reactions) | **Assembled**  ***P. veronii***  number (percent of library) | **Genome completeness** (percent) |
| --- | --- | --- | --- | --- | --- |
| **50-50 mock community** | **125** | **60 (48 %)** | **58 (97 %)** | **44 (76 %)** | **3.5 ± 2.6** |
| **10-90 mock**  **community** | **60** | **6 (10 %)** | **6 (100 %)** | **6 (100 %)** | **1.8 ± 1.3** |


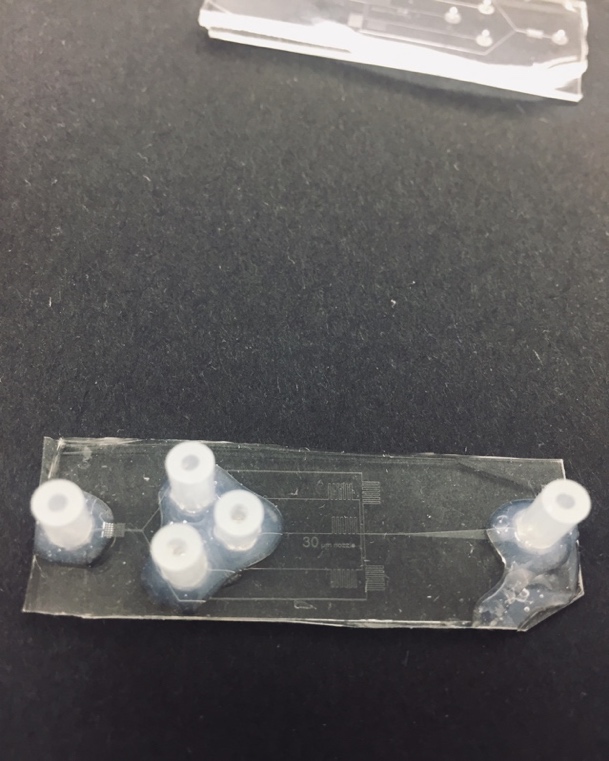

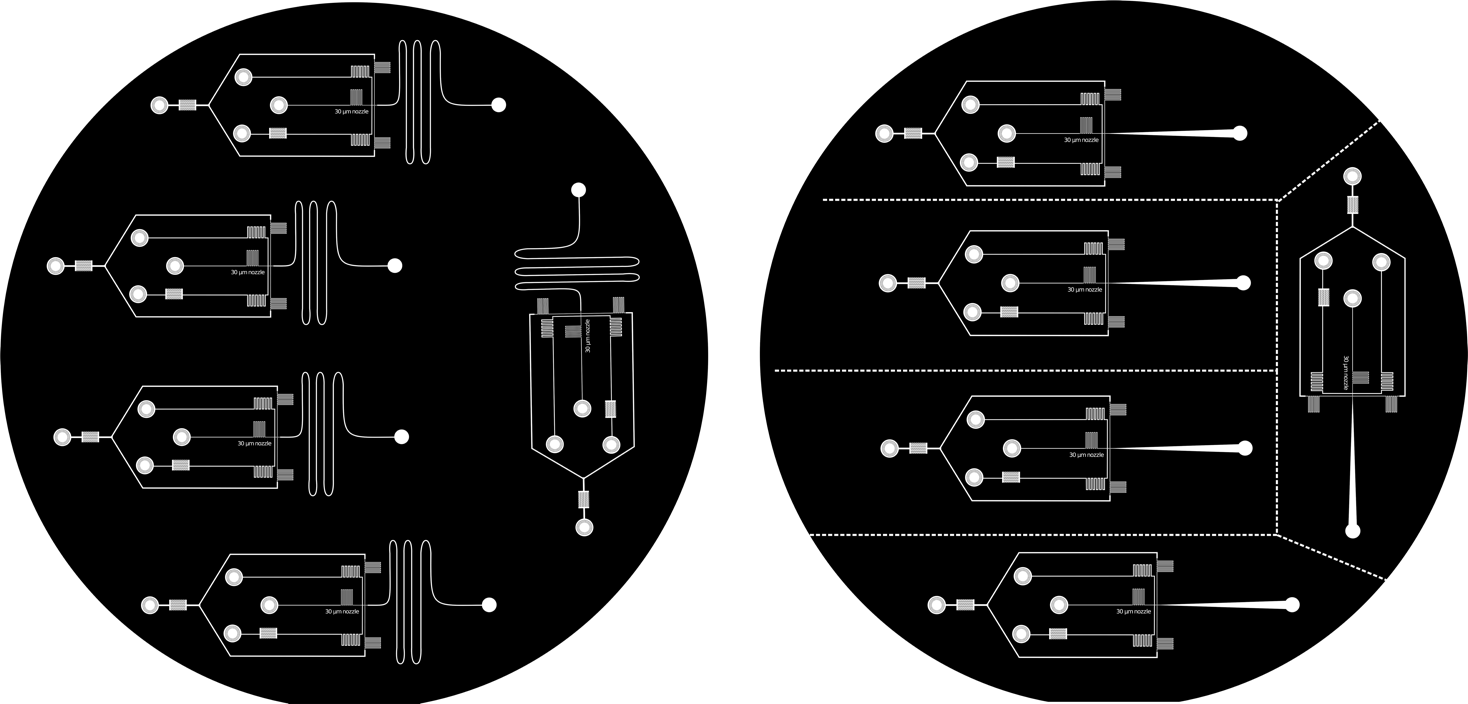


(C)

(B)

(A)

**Figure S1.** (A) The photomask for photolithography on a 4-inch silicon wafer and (B) the geometry details of the design pattern. (C) The microfluidic chip was fabricated in-house.

**Figure S2. The modified radiographic procedures for applying MAR on alginate microcapsules.** First, a 10 µm CellTrics cell strainer was placed on a clean 1.5 mL Eppendorf. All solutions including alginate microcapsules and the oil layer from the collection tube were transferred to the cell strainer, and then 180 µL of perfluorooctanol was evenly distributed over the filter to break the surfactant, which took approximately 1 minute. Next, the filter was carefully inverted and placed on a 24-well plate and backflush the alginate microcapsules from the filter into the 24-well plate with 400 µL DI water. Transferred all liquid from the 24-well plate to a clean 1.5 mL Eppendorf and centrifuged at 900 *g* for 3 minutes to separate the alginate microcapsules from the aqueous layer. After discarding the upper aqueous layer, the microcapsules were subsequently washed with DI water and centrifuged to remove the upper aqueous layer. 400 µL of developer was added to the MAR microcapsules and mixed thoroughly by pipetting gently up and down. The sample was incubated with the developer for 3 min before centrifugation at 900 *g* for 3 min. After discarding the supernatant, MAR microcapsules were washed twice with 400 µL DI water. Subsequently, 400 µL of fixer was added to the MAR microcapsules and mixed thoroughly by gently pipetting up and down. The sample was then incubated with the fixer for 3 min and centrifuged at 900 *g* for 3 min. After discarding the supernatant, the MAR microcapsules were washed twice with 400 µL DI water, re-suspended, and stored in 250 mM BaCl_2_ at room temperature until sorting.
